# From Precision Medicine to Precision Convergence for Multilevel Resilience—The Aging Brain and Its Social Isolation

**DOI:** 10.3389/fpubh.2022.720117

**Published:** 2022-07-05

**Authors:** Laurette Dubé, Patricia P. Silveira, Daiva E. Nielsen, Spencer Moore, Catherine Paquet, J. Miguel Cisneros-Franco, Gina Kemp, Bärbel Knauper, Yu Ma, Mehmood Khan, Gillian Bartlett-Esquilant, Alan C. Evans, Lesley K. Fellows, Jorge L. Armony, R. Nathan Spreng, Jian-Yun Nie, Shawn T. Brown, Georg Northoff, Danilo Bzdok

**Affiliations:** ^1^Desautels Faculty of Management, McGill Center for the Convergence of Health and Economics, McGill University, Montreal, QC, Canada; ^2^Ludmer Centre for Neuroinformatics and Mental Health, Douglas Mental Health University Institute, McGill University, Montreal, QC, Canada; ^3^Department of Psychiatry, Faculty of Medicine, McGill University, Montreal, QC, Canada; ^4^Faculty of Agricultural and Environmental Sciences, School of Human Nutrition, McGill University, Montreal, QC, Canada; ^5^Health Promotion, Education, and Behavior, Arnold School of Public Health, University of South Carolina, Columbia, SC, United States; ^6^Faculté des Sciences de l'Administration, Université Laval, Quebec City, QC, Canada; ^7^Centre for Research in Neuroscience, The Research Institute of McGill University Health Center, Montreal, QC, Canada; ^8^Department of Psychology, Faculty of Arts, McGill University, Montreal, QC, Canada; ^9^Life Biosciences Chief Executive Officer (CEO), Boston, MA, United States; ^10^Council on Competitiveness (Chairman of the Board), Washington, DC, United States; ^11^Department of Family Medicine, Faculty of Medicine, McGill University, Montreal, QC, Canada; ^12^Laboratory of Brain and Cognition, Department of Neurology and Neurosurgery, Montreal Neurological Institute, McGill University, Montreal, QC, Canada; ^13^Chronic Mental Illness Service, Montreal Neurological Institute, Montreal, QC, Canada; ^14^Douglas Mental Health University Institute, Montreal, QC, Canada; ^15^Departments of Psychiatry and Psychology, McGill University, Montreal, QC, Canada; ^16^McConnell Brain Imaging Centre, McGill University, Montreal, QC, Canada; ^17^Department of Computer Science and Operations Research, University of Montreal, Montreal, QC, Canada; ^18^Pittsburgh Supercomputing Center, Carnegie Mellon University, Pittsburgh, PA, United States; ^19^Faculty of Medicine, Brain and Mind Research Institute, University of Ottawa, Ottawa, ON, Canada; ^20^Department of Biomedical Engineering, Faculty of Medicine, McConnell Brain Imaging Centre (BIC), Montreal Neurological Institute (MNI), McGill University, Montreal, QC, Canada; ^21^Mila–Quebec Artificial Intelligence Institute, Montreal, QC, Canada

**Keywords:** social isolation, aging, precision convergence, primary care (MeSH), real-word behavior

## Introduction

Much like an engineering stress test, the successive waves of the COVID-19 pandemic have highlighted critical pressure points in our already distressed healthcare systems. Physical and mental health challenges continue to accumulate in non-infected persons (1) as well as in those suffering from long-lasting effects of the disease (2). Meanwhile, the implementation of physical distancing containment measures and the significant overload of healthcare systems have resulted in a drastic disruption of elective treatments for most chronic physical and mental diseases.

The pandemic has also emphasized the importance of precision medicine, a convergence between clinical, genomics, and computational sciences (3). A silver lining to this global crisis is the unique combination of precision medicine advances and an unprecedented collaboration between science, business, and society behind the rapid development of COVID-19 vaccines. In this *Opinion*, we argue that lessons from the COVID-19 vaccine response can inform our approach to other equally pressing health emergencies worldwide, such as the epidemic of loneliness in older adults.

We propose *precision convergence* as an approach to achieve this level of synergy in finding societal-scale real-world solutions for a resilient and healthier future, with health and healthcare systems at its core. Precision convergence extends and bridges the scientifically defined multi-scale mechanisms in biology and neuroscience on the one hand and social systems impacting *real-world behavior*, on the other. In this paper, we first elaborate on multi-level resilience and its links to precision convergence. We use social isolation among seniors as an exemplar, or test case, of how and why a precision convergence approach is necessary to potentiate transformative change.

## Precision Convergence for Multi-Level Resilience

Resilience refers to “the capacity of a system to tolerate disturbances while retaining its structure and function” (4). By *multilevel resilience*, we refer to the capacity, at every level of human endeavor—from individuals to professions, organizations and institutions of science and society at large—to adapt, evolve and grow in the face of challenging conditions and turbulent change that now define modern contexts.

**Figure 1:**
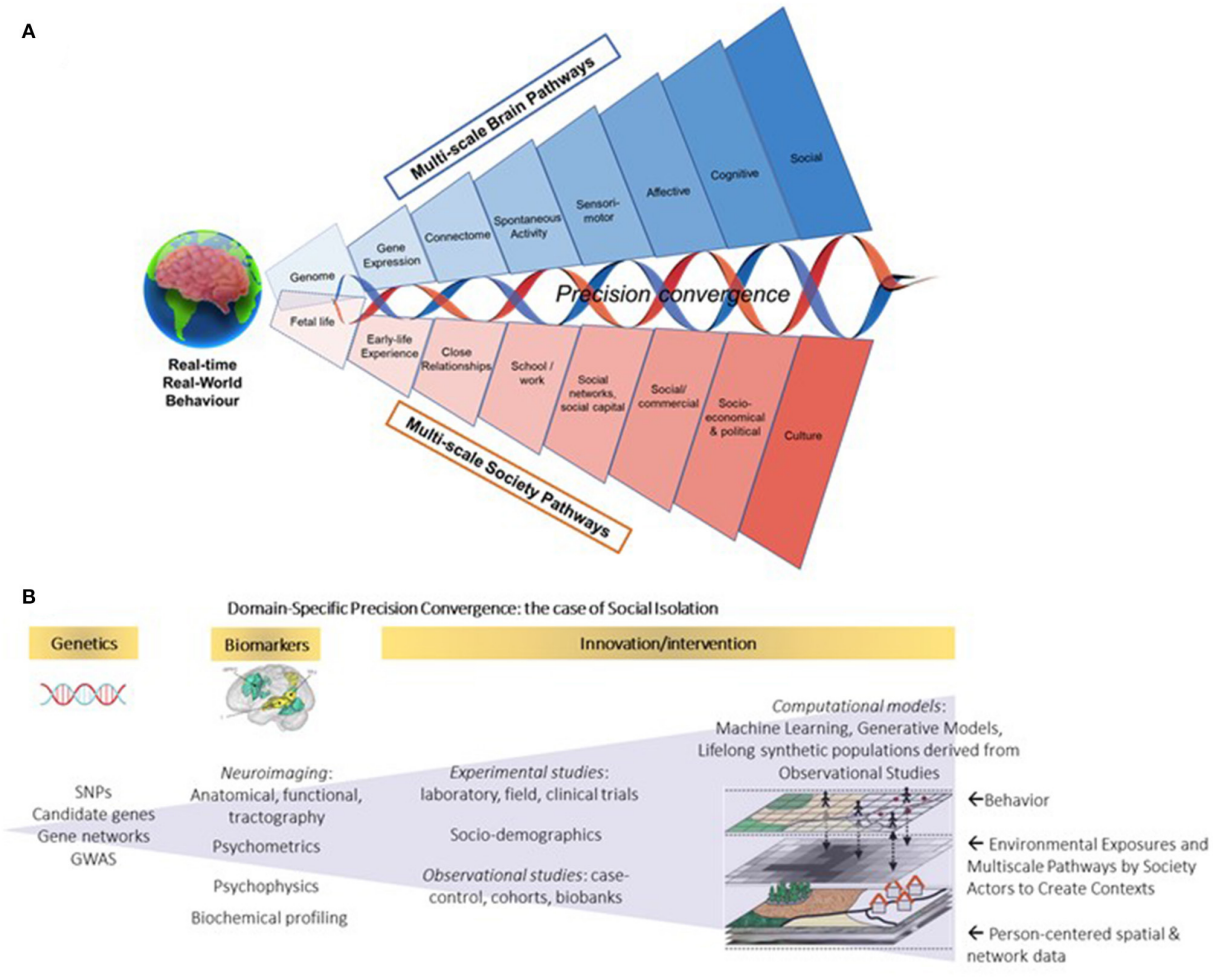
**(A)** Brain-to-Society (BtS) model of real-world behavior: lifelong achievement, wellbeing, and resilience. Real-world behavior is adaptive, responding to the demands of a changing environment in health and disease. **(B)** Domain-specific precision convergence for social isolation. By integrating multimodal data (e.g., genetics, environment, biometrics) from animal models, experimental studies and population-level research, Precision Convergence aims to strengthen circular linkages between science and action to innovate the way we innovate and inform real-world adaptive solutions toward building multi-level resilience. This figure has been designed in part using resources from Freepik.com (studiogstock, freepik, macrovector), with adaptations from Yang et al. (6) and Spreng et al. (7).

The speed of cultural evolution that marked the last two centuries is of a different geographical and temporal scale than biological evolution (5). Ironically, advances in economic wealth, social wellbeing, and public health interventions, have brought new challenges to human biology: with extended lifespans come chronic diseases, whereas food abundance and automation are associated with increasing obesity rates. In this context, individual choices are simultaneously conditioned by and powerful drivers of a system-level transformation in economy and society. Since these levels are interlocked, with each level entailing its own multiscale mechanisms, it is essential to understand the dynamics of such interactions as they evolve over a person's lifespan to inform decision making in the pursuit of a multilevel resilience that is not reducible to the sum of its parts (Figure 1A).

Multi-level resilience starts with the individual by *adaptive real-world behavior*, with everyday experience and behavior that support health and wellbeing in all dimensions (e.g., physical, psychological, social, cognitive, financial), while accounting for the diverse and dynamic roles and contexts along the lifespan. These are emergent properties of neurobiological systems that underlie one's reflexes, impulses, emotions, and cognitions, which are continuously weighted and re-weighted for adaptive behavior that ultimately guide resilience at an individual level (8). A closely related construct is that of cognitive reserve, defined as “the adaptability of cognitive processes that helps to explain differential susceptibility of cognitive abilities or day-to-day function to brain aging, pathology, or insult” (9). In this sense individual-level resilience stems from and in turn shapes a person's own life experience through the lifelong cumulation of risk and protective factors (10). From this perspective, understanding the human-defining features of brain and mind is a key lever for adaptive, real-world behavior in response to the environmental context created by the economy and society. The COVID-19 pandemic may have fostered resilience at an individual level by forcing a better balance between goals, aspirations, expectations, and achievement of individuals in our roles as parents, citizens, workers or patients vis-à-vis consumer roles, shifting in favor of a more value-driven and meaningful life.

Resilience at the level of professions, organizations, and institutions requires decision makers to be risk-aware, flexible, and agile for real-time and long-term performance. Strategies to reach economy- and society-level resilience have yet to fully create *adaptive* real-world contexts for individuals. Cross-disciplinary and cross-sectoral action focused on target domains of behavioral change and ecosystem transformation are needed, positioning the person at the center of this complex adaptive system of systems in constant evolution (Figure 1B). Better accounting for complex and dynamic multi-scale interdependencies is at the core of multi-level resilience and precision convergence.

Precision Convergence is an integrative governance framework that embeds ethical, social and privacy concerns into science, technology, innovation, and policy. Propelled by the digitization of science and society, the vision of precision convergence is to weave next generation biomedical sciences, technologies, processes, and devices needed for real-world solutions in specific domains. Its purpose is to reimagine research, technology, innovation, and policies that better support multi-scale resilience in our transforming world, motivating circular linkages between science and action to *innovate the way we innovate*. This requires a person-in-system approach with a digital-powered and human-centered focus on individual and system-level solutions (11), while acknowledging the challenges and possibilities tied to the multiscale (temporal, spatial or socio-political) mechanisms operating within and across levels in creating adaptive real-world contexts.

## Social Isolation and the Aging Brain: A Test-Case for Precision Convergence

Protective lifestyle factors that may contribute to increased individual resilience and cognitive reserve include physical exercise, education, and engaging in cognitive and social activities (12), with a recent meta-analysis showing that social isolation was associated with impaired late-life cognitive function (13). As people grow older, their social network typically becomes smaller and oftentimes also weaker (14), with dramatic consequences for individual resilience by impacting brain and physical health (15, 16). This necessary source of interpersonal stimulation has been severely disrupted throughout the COVID-19 pandemic, with a significant weakening or complete loss of social connections within communities. In recognition of the magnitude of this issue pre-COVID-19, the World Health Organization have declared an epidemic of loneliness and the UK have appointed a “Minister of Loneliness” and initiated a campaign encompassing 600 national, regional, and local organizations to reduce loneliness in later life. More recently, Japan followed suit in appointing a Minister of Loneliness to tackle heightened suicide rates linked to COVID-19. Translating these into real-world transformation engaged multiple professions, organizations, and institutions in health and other sectors.

Another concurrent development is our increased ability to gather, structure, and analyze more significant quantities of data with greater efficiency than ever. Propelled by artificial intelligence, big-sample datasets on microanatomy, multi-scale synaptic connections, optogenetic brain-behavior assays, and high-level cognition, research in neurosciences has transformed our understanding of adaptive real-time decisions and behavior (17).

Combined with the digital transformation of science and society, it is possible to support solution-oriented science, technology, innovation, and policy, enabling multiscale-resilience. Consider, for instance, the UK Biobank, the premier “big data” resource that links genotyping, phenotyping and contextual information on ~500,000 individuals, making it possible to overcome this unbridgeable brain-to-society gap. This is now enabling researchers to investigate complex gene-by-environment interactions underpinning individual differences in brain structure and cognitive processes to better understand and predict health/disease pathways and societal-level behaviors. A recent example of this is our investigations of key brain networks underlying sociality (15) and their functional and structural shifts that are associated with perceived social isolation (7, 16). These studies have enhanced our understanding of how the structure and function of the human brain has led to the evolution of humans as the “ultimate social animal.”

These developments in societal structures, policy and technology allow us to investigate social isolation through the lens of precision convergence, building on fundamental research findings in animal models, from gene regulation to the neurophysiological and anatomical correlates of social isolation to building synthetic simulations of environmental factors (Figure 1B). Considering that the relative contribution of genetics and/or social determinants of health may vary across the lifespan, Precision Convergence recognizes the diversity of biological predispositions and psychological trajectories in terms of experiences and contexts in the elderly. That, in turn, influences how social network disruptions affect brain structure and function, and how such disruptions influence society. By integrating real-world data across studies (e.g., discovery cohorts, biobanks), currently available and continuously improving computational models can be used to derive representative synthetic populations and develop simulations to test *in silico* policy interventions to support resilience across all levels of society.

## Precision Convergence and Medicine in Action

Throughout the COVID-19 pandemic, the lack of resilience in global healthcare systems has been laid bare and we argue that this stems from a lack of convergence between health and other sectors of society. Countries around the world cannot afford to sustain the current tertiary-centered health care system where research and operational investments have resulted in shrinking budgets for primary care or public health initiatives.

Primary care is often a critical intermediary between medicine, public health, and other agents in the community, thereby contextualizing patient needs against the sociocultural backdrop of the world in which they live. This exemplifies the benefits of a precision convergence-guided approach. The personalization of medicine—unique to primary care—rests on an intimate understanding of a community's social and cultural fabric. Given that COVID-19 is impacting people and communities differently, these defining characteristics of primary care need to be supported and strengthened, transforming front line providers into catalysts for change throughout society. This may be achieved by applying Precision Convergence to the evolution of primary care to encompass a team-based approach providing support in all relevant domains: physical, psychological, cognitive, social, and financial. This is already being implemented through the concept of “social prescription” where primary physicians refer patients to community-based support entities (18).

The importance of primary care in improving resilience cannot be understated, serving as a bridge between science and action by individuals, health systems and society. Case in point, obesity, diabetes and heart conditions are established risk factors for developing complications from COVID-19 infections (19). Therefore, improving the metabolic health of populations may assist in building resilience against future infectious disease outbreaks. Moving forward, applying the person-centered approach at the core of Precision Convergence will be essential in reshaping healthier environments. Further, technology-enabled care that the pandemic has forced to the forefront of primary care can help manage routine care by embedding innovations into everyday life as seen with virtual clinical visits.

The accelerated adoption of telehealth, however, has highlighted deficits in our digital infrastructure (20). Increasing accessibility to hardware, software and internet to patients and clinicians alike is important for equitable access in preparation for future public health emergencies (21). Not only will this have implications for access to technology-enabled care, but it also has important repercussions for employment, social cohesion, education, and the environment. This will be critical to a human-centered, digitally powered approach to support health and a recovering economy, including safety monitoring, adherence to vaccination schedules, and tracking population-level vaccination and immunity rates.

Finally, it is necessary to rethink the training of our health care workforce, researchers, policy makers as well as others in all the related domains linked in a convergence approach that is anchored in complexity science and person-centered societal transformation. Going forward, a stronger orientation toward primary care and a precision convergence approach may prove useful in strengthening our healthcare systems to heal from the COVID-19 pandemic and to withstand the impacts of the next global health crisis.

## Conclusion

Precision convergence builds on longstanding recommendations for human-centered convergence between biomedical sciences (3), policy and action in social, health, and economic domains (22) as well as on more recent calls for solution-oriented social (23) and computational social sciences (24). Our species has reached its current state by adapting to ever more complex group dynamics in families, communities, nations, as well as social institutions and globe-spanning digital communities. The time may be ripe to change the way science and society have operated for centuries. This will require working toward a new era of multi-level resilience that better accounts for interdependencies and connections between individual real-world behaviors and population-level environmental, social, and economic contexts. We believe that harnessing multi-level resilience is possible in a post COVID-19 world and that precision convergence with health sciences at its core can and must be catalysts of such transformation.

## Author Contributions

LD: conceptualization and project administration. LD, JC-F, GK, and GN: visualization. LD, JC-F, GK, GN, and DB: writing—original draft. LD, PS, DN, SM, CP, JC-F, GK, BK, YM, MK, GB-E, AE, LF, JA, RS, J-YN, SB, GN, and DB: writing—review and editing. All authors contributed to the article and approved the submitted version.

## Funding

This work was supported in part by the National Institutes of Health (NIH R01 230 AG068563 to RS and DB), the Social Sciences and Humanities Research Council (SSHRC Grant # 895-2019-1011 to LD), and a Healthy Cities Training Grant (Tri-Agency fund: CIHR-NSERC-SSHRC # 020863 - 000 / 0609003969 to LD, CP, JC-F, YM, and PS).

## Conflict of Interest

The authors declare that the research was conducted in the absence of any commercial or financial relationships that could be construed as a potential conflict of interest.

## Publisher's Note

All claims expressed in this article are solely those of the authors and do not necessarily represent those of their affiliated organizations, or those of the publisher, the editors and the reviewers. Any product that may be evaluated in this article, or claim that may be made by its manufacturer, is not guaranteed or endorsed by the publisher.
